# Improving Stability and Mechanical Strength of Electrospun Chitosan‐Polycaprolactone Scaffolds Using Genipin Cross‐linking for Biomedical Applications

**DOI:** 10.1002/marc.202400869

**Published:** 2024-12-27

**Authors:** Nagalekshmi Uma Thanu Krishnan Neela, Piotr K. Szewczyk, Joanna E. Karbowniczek, Martyna Polak, Joanna Knapczyk‐Korczak, Urszula Stachewicz

**Affiliations:** ^1^ Faculty of Metals Engineering and Industrial Computer Science AGH University of Krakow al. A. Mickiewicza 30 Krakow 30–059 Poland

**Keywords:** chitosan, electrospinning, genipin, mechanical property, polycaprolactone

## Abstract

Electrospun nanofiber scaffolds have become vital in biomedical applications due to their high surface area and tunable properties. Chitosan (CS) is widely used, but its rapid degradation limits its effectiveness. This study addresses this limitation by blending CS with polycaprolactone (PCL) and applying genipin cross‐linking to enhance its stability and mechanical properties. Scanning electron microscopy indicated a uniform morphology of the electrospun fibers, and further, the crystallinity of the scaffolds before and after cross‐linking is verified. Fourier‐transform infrared spectroscopy is used to analyze the chemical structure, identifying the presence of trifluoroacetic acid residues in the as‐spun fibers. These residues are successfully eliminated through neutralization and cross‐linking, which are critical for enhancing stability and cell viability in in‐vitro studies. Mechanical testing revealed that cross‐linked CS+PCL scaffolds exhibit a 350% increase in tensile strength compared to pure CS, and zeta potential reaches the favorable for cell development ‐26.27 mV. The cytotoxicity assay results with murine NIH 3T3 fibroblast cells indicate the suitability of CS+PCL scaffolds for targeted tissue engineering and wound healing. This work establishes the potential for fine‐tuning scaffold properties to create stable, functional, and biocompatible substrates for extended biomedical use.

## Graphical abstract

Chitosan (CS) is a natural polymer with numerous advantages in biomedical applications. However, its poor mechanical strength and rapid biodegradation are significant limitations. Its properties are improved by blending with polycaprolactone (PCL) and genipin cross‐linking. It results in a 350% increase in tensile strength compared to pure CS scaffolds along with significantly higher stability and biocompatibility.

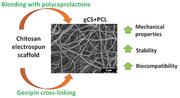

## 1 Introduction

In recent years, advancements in tissue engineering, drug delivery, and wound healing have been significantly driven by nanotechnology.^[^ ^1^, ^2^ ^]^ Electrospinning enables the creation of nanofiber scaffolds with high surface area and tunable physicochemical properties, including fiber diameter,^[^ ^3^, ^4^ ^]^ wettability,^[^ ^5^ ^]^ degradation profile,^[^ ^6^ ^]^ mechanical strength,^[^ ^7^ ^]^ and surface charge.^[^ ^8^, ^9^ ^]^ Many parameters are crucial to obtaining the desired properties of electrospun fibers, including voltage polarity, humidity, and more.^[^ ^10^, ^11^, ^12^, ^13^, ^14^ ^]^

These features make electrospun materials highly suitable for biomedical applications such as drug delivery systems,^[^ ^15^ ^]^ skin patches,^[^ ^16^ ^]^ wound dressings,^[^ ^17^, ^18^ ^]^ and cell scaffolds.^[^ ^19^ ^]^ Natural polymers like chitosan (CS), collagen, gelatin, elastin, and silk fibroin are often used in electrospinning materials for biomedical applications due to their biocompatibility, biodegradability, and ability to support cell migration and proliferation.^[^ ^20^ ^]^However, these polymers are frequently blended with synthetic ones to improve mechanical stability and processability, achieving enhanced spinning properties and tailored degradation rates essential for targeted applications.^[^ ^21^ ^]^

CS is a biocompatible, biodegradable polymer with low toxicity, making it desirable for biomedical research.^[^ ^20^ ^]^ Structurally, CS is a polycation (under its pKa) composed of functional units of the amino (NH_2_) and the hydroxyl (‐OH) groups and is a linear polysaccharide obtained by the deacetylation of chitin.^[^ ^22^ ^]^ CS is known for its excellent properties and enhanced cell‐material interactions, and widely used in bone, skin, and blood vessel tissue engineering, drug delivery systems, and periodontal and corneal regeneration.^[^ ^23^, ^24^, ^25^ ^]^ However, the use of CS is limited as it dissolves in various aqueous solutions, as well as bodily fluids like saliva.^[^ ^26^, ^27^ ^]^ The limitation can be overcome by modification of CS by neutralizing agents and crosslinkers.^[^ ^28^, ^29^, ^30^ ^]^

The practice of cross‐linking individual macromolecules through covalent or non‐covalent bonds has gained significant attention in recent years. This technique involves creating a 3D network by bonding molecules together.^[^ ^31^ ^]^ Such networks enhance the stability, strength, and structural integrity of various materials. Additionally, cross‐linking can stabilize a polymer's structure, allowing for the formation of more defined shapes. Agents such as glutaraldehyde, epoxides, and natural linkers like genipin are commonly used for this purpose. While glutaraldehyde is effective, it can have cytotoxic effects, making natural cross‐linkers like genipin more appealing for biocompatible applications. Recent studies highlight the advantages of natural cross‐linkers in maintaining biodegradability and enhancing the mechanical properties of the materials.^[^ ^32^ ^]^ The chemical cross‐linking with genipin, a natural cross‐linker, is widely studied due to its noncytotoxic characteristic in contrast to glutaraldehyde.^[^ ^33^ ^]^ The genipin helps to improve the mechanical properties depending on the concentration. It allows the electrospun scaffolds to maintain their morphology and controls the degradation dynamics of the scaffold.^[^ ^34^, ^35^ ^]^ Genipin cross‐links chitosan effectively, improving its strength, stability, biocompatibility, and biodegradability for medical use, as it decreases inflammation and oxidative stress, making it a good choice for wound healing applications.^[^ ^35^ ^]^ Its low toxicity and ability to support cell growth make it ideal for tissue engineering and regenerative applications.^[^ ^33^ ^]^

Polycaprolactone (PCL) is a synthetic, semicrystalline polyester extensively researched in biomedical applications, including wound healing and tissue engineering.^[^ ^36^ ^]^ PCL is known for its excellent biocompatibility, biodegradability, and mechanical properties, making it a versatile material blended with other polymers.^[^ ^36^, ^37^ ^]^ Its slow degradation rate allows for extended support in tissue regeneration, while its mechanical strength enhances scaffolds' structural integrity.^[^ ^38^ ^]^ Blending PCL with CS can overcome chitosan's mechanical limitations while maintaining its biocompatibility and biodegradability, making the combination highly suitable for developing functional scaffolds for skin tissue engineering.

In this study, we utilized genipin to cross‐link CS and its blend with PCL and investigated how cross‐linking and polymer blending impact the resulting scaffolds. We aim to improve the mechanical performance and stability of chitosan‐based scaffolds in cell culture studies. Our findings provide insight into how these modifications can enhance the properties of chitosan‐based materials, making them more suitable for drug delivery systems and tissue engineering applications. Additionally, one of the possible applications of CS materials is wound dressings, which are exchanged every day; therefore, their prolonged stability is not required, and reported in the manuscript 3 days of stabilization achieved by blending CS with PCL and genipin crosslinking is sufficient. The results demonstrate the potential for tailoring scaffold properties to improve their functionality in biomedical applications.

## 2 Results and Discussion

### 2.1 Morphology of Prepared Scaffolds

**Figure 1:**
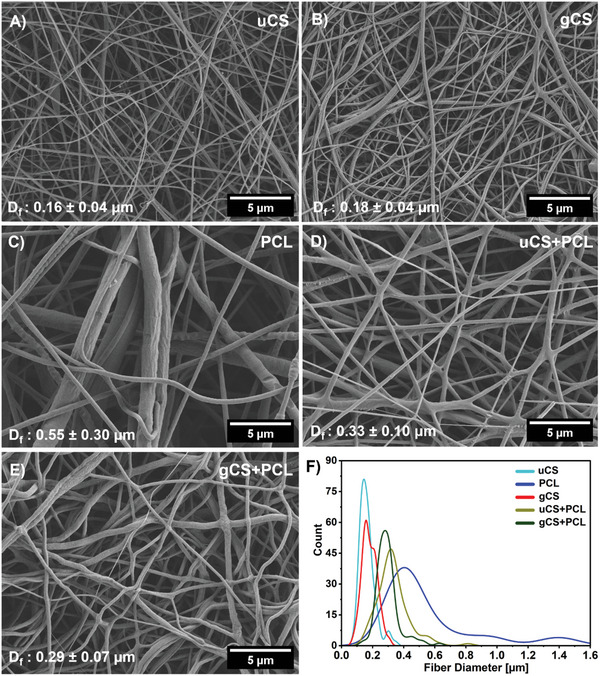
SEM micrographs of A) uCS, B) gCS, C) PCL, D) uCS + PCL, E) gCS + PCL, and F) Fiber diameter distribution graph (Gaussian fit) for all electrospun samples.

The morphology and structural characteristics of uncross‐linked and cross‐linked electrospun nanofibers were analyzed using scanning electron microscopy (SEM) imaging, as shown in **Figure** **1**. Several studies have reported the optimization of PCL to CS ratio for electrospinning and they have stated that the equal ratio of PCL to CS can produce fibers reproducible at every time with similar fiber diameter and spinnability. Based on the insight from these studies, we have chosen this ratio of PCL to CS.^[^ ^39^ ^]^ The uncross‐linked fibers of chitosan (uCS), polycaprolactone (PCL), and chitosan+polycaprolactone (uCS+PCL) exhibited smooth surfaces without pores or beads, with average diameters of 0.16 ± 0.04 µm, 0.55 ± 0.30 µm, and 0.33 ± 0.10 µm, respectively. After cross‐linking with genipin, the fiber morphology remained unchanged, although we observed slight differences in fiber diameter. The specific values were 0.18 ± 0.04 µm and 0.29 ± 0.07 µm for genipin crosslinked fibers of the chitosan (gCS), and genipin cross‐linked fibers of the chitosan+polycaprolactone (gCS+PCL), respectively. The cross‐linked scaffolds gCS and gCS+PCL maintained smooth fiber morphology without aggregation of fibers (Figure 1). We have found that electrospinning of CS and CS+PCL from TFA is possible as it interacts with CS amino groups, disrupting rigid molecular interactions.^[^ ^40^ ^]^ However, PCL is not involved in the cross‐linking with genipin due to lack of amino groups.^[^ ^41^ ^]^ The drawback of such an approach is that uncross‐linked CS and CS+PCL electrospun scaffolds lose their fibrous structure rapidly in aqueous environments, making cross‐linking essential to maintain fiber integrity in such conditions. Thus, genipin, a common cross‐linker, was used to stabilize the fibers, as described in the materials section.^[^ ^33^ ^]^ At the same time, pure PCL remained hydrophobic, with a contact angle of 123° ± 1.6°, reaching values in the range previously reported.^[^ ^36^ ^]^ For the samples containing CS, the water was observed to rapidly seep into the scaffolds upon placement. As a result, the water contact angle for uCS, gCS, uCS+PCL, and gCS+PCL could not be measured due to the immediate absorption of the water droplet, see Figure S1 and Video S1 (Supporting Information) illustrating this behavior. Similar reports have been mentioned in earlier studies as well.^[^ ^42^, ^43^ ^]^ The obtained results indicate that the morphology of the electrospun fibers remained consistent after cross‐linking with genipin without significantly altering their structural and wetting characteristics. In Figure S2A–E (Supporting Information), the electrospun scaffolds, including uCS, gCS, uCS+PCL, gCS+PCL, and PCL, along with the genipin cross‐linked scaffolds are presented. The images of electrospun scaffolds before and after 0.25% genipin cross‐linking show color changes, indicating successful cross‐linking. Figure S2B–D, Supporting Information) shows genipin cross‐linked scaffold exhibiting a pale green color.^[^ ^44^ ^]^

### 2.2 Chemical and Structural Analysis of Scaffolds

**Figure 2:**
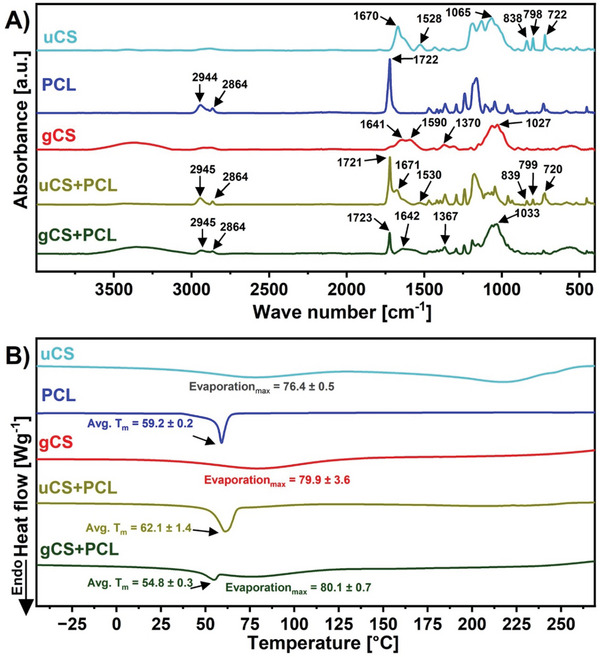
A) FTIR and B) DSC analysis of all the samples: uCS, PCL, gCS, uCS+PCL, and gCS+PCL fibers.

Fourier transform infrared spectroscopy (FTIR) analysis was conducted to verify the materials' chemical composition, and the FTIR spectra are shown in **Figures** **2**A and S3 (Supporting Information). The CS flakes used as a reference sample gave characteristic peaks for the amide and CH_2_OH groups at 1654 and 1023 cm^−1^ (Figure S3, Supporting Information). Which are within the range reported in the literature.^[^ ^46^ ^]^ In uCS, the responsible peak of amide I was found at 1670 cm^−1^, amide II peak at 1528 cm^−1^, and C═O groups at 1065 cm^−1^ (Figure 2A). The shift in the characteristic peaks in uCS is caused by electrospinning and the use of TFA as a solvent.^[^ ^45^ ^]^ TFA influence is further confirmed by peaks at 838, 798, and 722 cm^−1^, which correspond to the trifluoroacetate anions, which are caused by the TFA‐based salt formation in the CS backbone after dissolving the CS in TFA.^[^ ^46^ ^]^This confirms residual TFA in the structure after fiber preparation. For the pristine PCL pellet and PCL fibers, we have observed a characteristic peak around 1722 cm^−1^, tied to an ester bond (RCOOR′) (Figure 2A; Figure S3, Supporting Information). This value is also reported in the literature.^[^ ^47^ ^]^After blending uCS and PCL, the sharp overlapping peak at 1671 cm^−1^ (amide I from CS) near 1721 cm^−1^ (RCOOR′ from PCL) was identified with other TFA‐related peaks at 839, 799, and 720 cm^−1^ (trifluoroacetate anions), which is indicated in Figure 2A. This confirms that the TFA is present in the structure even in the blended state. However, upon neutralizing uCS and uCS+PCL with NaOH and methanol, the TFA‐specific groups are deprotonated by ‐OH ions. This reaction removes the TFA salt associated with the ammonium (─NH₃⁺) groups in CS, indicating that they are converted back to amine (─NH₂) groups.^[^ ^46^ ^]^It is confirmed on the samples after neutralization and cross‐linking with genipin (gCS+PCL and gCS), where the peaks at 720–840 cm^−1^ vanish, proving that genipin stabilizes the structure and can purge TFA from the material (Figure 2A).

We confirmed the cross‐linking of gCS by the presence of characteristic peaks at 1590 cm^−1^ (C═N stretching), 1641 cm^−1^(amide I), 1370 cm^−1^ (CH_2_ groups), and 1027 cm^−1^ CH_2_OH, which are slightly shifted by close to the literature values which are 1595, 1635, and 1027 cm^−1^ for C═N stretching, amide I and CH_2_OH (Figure 2A).^[^ ^46^, ^48^, ^49^, ^50^ ^]^Peaks correlated with TFA are absent after neutralization and cross‐linking, confirming that our preparation technique is also suitable for blended polymers.^[^ ^51^ ^]^

The scaffolds' structure was analyzed using differential scanning calorimetry (DSC). The first heating cycle was used to assess the scaffolds' structure post‐electrospinning. Figure 2B presents the thermograms for uCS, PCL, gCS, uCS+PCL, and gCS+PCL, while Figure S4 (Supporting Information) shows the thermographs of CS flakes and PCL pellets used to prepare the electrospinning solutions. For samples containing CS, results were compared with those of CS flakes, which displayed an endothermic peak at 80.8 ± 0.6 °C. This peak corresponds to the evaporation of water bound to the hydrophilic ─OH and ─NH_2_ groups in CS, highlighting the strength of water‐polymer interactions.^[^ ^52^, ^53^ ^]^ Similar peaks associated with water evaporation were observed in uCS, gCS, and gCS+PCL. However, in electrospun fibers, the water evaporation peaks shifted to lower temperatures, with maxima at 76.4 ± 0.5 °C, 79.9 ± 0.5 °C, and 80.1 ± 0.7 °C, respectively, likely due to the high surface area‐to‐volume ratio of the electrospun materials.^[^ ^54^ ^]^

The degree of crystallinity in PCL was investigated and compared to the PCL pellets used for electrospinning. The crystallinity of electrospun PCL was 51.2 ± 0.6%, lower than the pellet's 53.4 ± 1.5%. This reduction aligns with previous studies linking lower crystallinity to rapid solvent evaporation during electrospinning, which limits the rearrangement, nucleation, and crystallization of PCL molecular chains.^[^ ^55^ ^]^Further reductions were observed in blended samples: uCS+PCL and gCS+PCL exhibited crystallinity values of 48.8 ± 0.3% and 10.3 ± 0.7%, respectively. These findings are consistent with reports showing that increased CS content in blends reduces the melting temperature and crystallinity of PCL. Moreover, very low crystallinity after crosslinking implies that strong cross‐linking reduces the crystallinity of PCL. Uto et al. reported almost completely amorphous PCL films when benzoyl peroxide was introduced.^[^ ^56^ ^]^ Blended samples did not exhibit any new peaks compared to pure CS and PCL, confirming the miscibility of the two polymers. Similar observations were reported by Bhattarai et al.^[^ ^57^ ^]^ Additionally, the broadening of the PCL melting peak upon CS introduction suggests a decrease in PCL crystalline integrity due to miscibility, as similarly noted by Han et al. for PCL cross‐linked with benzoyl peroxide.^[^ ^58^, ^59^ ^]^ It can also mean a variation of the ordered structures within the fibers, as reported by Baptista et al.^[^ ^60^ ^]^ A notable difference in the thermograms of uCS+PCL and gCS+PCL is the presence of a water evaporation peak in gCS+PCL, which is absent in uCS+PCL.^[^ ^60^, ^61^ ^]^ The observed difference is likely attributed to the cross‐linking process, which involves immersing the fibrous scaffolds in aqueous media during rinsing. This step reintroduces water into the fibers, where it binds to the hydrophilic groups of CS and becomes integrated into the structure.

### 2.3 Mechanical testing

**Figure 3:**
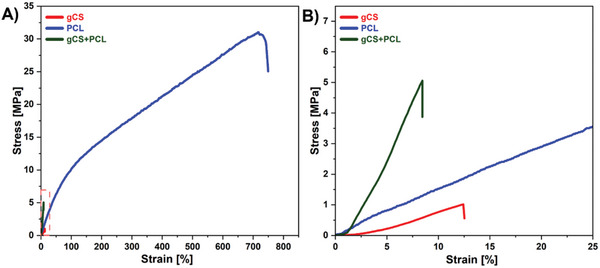
Stress‐strain curves from the tensile test for A) gCS, PCL, gCS+PCL electrospun scaffolds with B) Magnified section between 0 and 25% strain shown as red square.

Mechanical stability is a critical factor for scaffolds in biomedical applications.^[^ ^62^ ^]^ To evaluate this, we conducted tensile tests on our samples. **Table** **1** summarizes the results of tensile tests for all samples: maximum stress, maximum strain, elongation at failure, and toughness. The typical stress‐strain curves are shown in **Figure** **3**, and all results from tensile tests are presented in Figure S5A–E (Supporting Information). uCS electrospun scaffold had a maximum stress of 3.4 ± 0.2 MPa, maximum strain of 25.6 ± 4.1%, elongation at failure of 27.2 ± 2.3% and toughness of 61.0 ± 3.9 Jm^−3^. The uCS scaffold has low mechanical strength because of kinematic resistance resulting from hydrogen bonding and a wide range of cyclic structures in the molecular structure.^[^ ^63^, ^64^ ^]^ As expected, the PCL nanofibers exhibited much higher mechanical properties than uCS, such as 32 times higher maximum strain and 10 times higher maximum stress. Compared to uCS, uCS+PCL had a higher maximum stress of 5.8 ± 1.0 MPa. However, the maximum strain, elongation at failure, and toughness values were lower than for the uCS. The brittle nature of uCS leads to a decrease in the elasticity of the uCS+PCL scaffold, which results in lower elongation at failure values.^[^ ^63^, ^65^, ^66^ ^]^ After genipin cross‐linking, the gCS and gCS+PCL samples showed lower maximum stress, maximum strain, elongation at failure, and toughness compared to the uCS and uCS+PCL. Genipin cross‐linking stabilizes chitosan by reducing its solubility and enhancing its structural integrity when combined with PCL.^[^ ^41^ ^]^ This synergistic effect results from enhanced compatibility and load distribution between the chitosan and PCL phases, reduced phase separation, and increased aqueous stability. Thus, showing the stability of gCS+PCL in aqueous conditions. Notably, the gCS and gCS +PCL exhibit “J‐shaped” stress‐strain curves (Figure 3; Figure S5A–D, Supporting Information), and some previous studies show that these curves represent biological tissues, such as ligaments, skin, blood vessels, etc.^[^ ^67^, ^68^ ^]^A distinct stress‐strain behavior observed in various polymers and biological tissues defines the J‐shaped mechanical characteristic. The phenomenon matches closely with the native skin, where the tensile strength is between 5–30 MPa, and the gCS+PCL lies in that range.^[^ ^69^ ^]^ This unique mechanical behavior, not typical in polymers, allows for high stretchability while minimizing damage. Moreover, this characteristic suggests that gCS+PCL scaffolds could mimic the mechanical properties of native tissues, enhancing their suitability for load‐bearing tissue engineering applications.

**Table 1:** Tensile test results of uCS, gCS, PCL, uCS+PCL, and gCS+PCL electrospun fibers with the values of the maximum stress, maximum strain, strain at the maximum stress, and toughness. Errors are based on standard deviation, with *N* = 3, where N is the tensile test of one mat per sample.

Samples	Maximum stress [MPa]	Strain at maximum stress [%]	Maximum strain [%]	Toughness [Jm^−3^]
uCS	3.4 ± 0.2	25.6 ± 4.1	27.2 ± 2.3	61.0 ± 3.9
gCS	1.6 ± 0.5	10.1 ± 2.8	10.3 ± 2.8	6.5 ± 18.6
PCL	31.9 ± 3.1	801.7 ± 90.2	834.2 ± 90.7	16 607.1 ± 2182.1
uCS+PCL	5.8 ± 1.0	14.9 ± 1.8	16.2 ± 1.7	55.6 ± 11.4
gCS+PCL	5.4 ± 1.1	7.7 ± 1.3	7.8 ± 1.3	16.9 ± 2.5

### 2.4 Zeta potential

Zeta potential (ζ) is a key to understanding the interaction between the scaffold and electrolytes that flow into the porous architecture of the electrospun scaffold and the ions that interact with the scaffold's surface; this interaction is measured via the streaming potential.^[^ ^36^ ^]^ The zeta potential of gCS and gCS+PCL scaffolds was measured with a solution that provided constant ionic conditions that mimicked physiological ones at a pH of 7.^[^ ^36^ ^]^ The zeta potential was not evaluated for uCS and uCS+PCL samples due to scaffold degradation and swelling upon contact with water in an aqueous environment. Consequently, zeta potential measurements were conducted only on the cross‐linked samples. The gCS scaffolds exhibited a zeta potential of −13.66 ± 0.30 mV, higher than the gCS+PCL fibers, for which the value was −26.27 ± 0.03 mV. This indicates stronger interaction with ions for the gCS + PCL sample. The zeta potential for both samples is negatively charged and in the range suitable for biomedical applications such as drug delivery and tissue engineering.^[^ ^70^, ^71^, ^72^ ^]^

### 2.5 Indirect cytotoxicity and scaffold degradation

**Figure 4:**
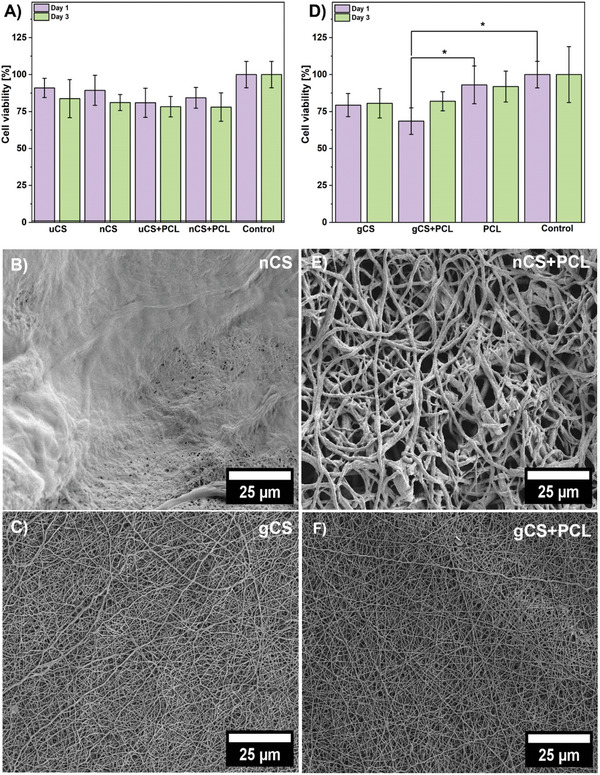
The cell cytotoxicity test results performed with NIH 3T3 cells for A) uCS, nCS+PCL, uCS+PCL, nCS+PCL, SEM micrographs of the scaffold after 3 days of incubation in the cell culture medium B) nCS and C) gCS, D) Cytotoxicity results for gCS, PCL, SEM micrographs of the scaffold after 3 days of incubation in the cell culture medium: E) nCS+PCL and F) gCS+PCL. ^*^Statistical significance was calculated with ANOVA, followed by Tukey's post‐hock test, *p* < 0.05; error bars are based on standard deviation.

The indirect cytotoxicity assay assesses the initial biocompatibility of scaffolds by verifying if any toxic substances are released. In this study, a cell titer blue assay was performed to assess cell attachment, while cell viability was measured by detecting cell metabolic activity. The metabolic capacity of cells was indicated by the fluorescent signal generated from the reduction of resazurin to resorufin, measured fluorometrically. These experiments were conducted using 3T3 fibroblast cells, with extracts collected after incubating the scaffolds in a cell culture medium for 1 and 3 days. **Figure** **4**A shows for uCS scaffolds, 91% cell viability while exposed to extracts from 1‐day incubation and dropped to 84% when extracts after 3 days were used. This indicates reduced viability caused by the leaching of TFA salts from the scaffold, which fully dissolved over time (see Figure S6A, Supporting Information). In contrast, when exposed to extracts from neutralized chitosan (nCS), cell viability exhibited 89% and 81% for days 1 and 3, respectively. The viability decrease most likely resulted from scaffold degradation and changes in the microenvironment, as confirmed by partially degraded fibers shown in Figure 4B. Neutralization slowed fiber degradation compared to pristine fibers and eliminated residual TFA, stabilizing the structure (Figure S6B, Supporting Information). Extracts from gCS, which did not have TFA in the structure, demonstrated a stable effect on cell viability of 79% for day 1 and 80% for day 3, indicating a maintained scaffold structure and low cytotoxicity (Figure 4C,D). Cell viability while exposed to extracts from PCL scaffolds was very high: 93% for day 1 and 92% for day 3, indicating stable biocompatibility, consistent with previous studies.^[^ ^36^ ^]^Extracts from blend uCS+PCL scaffolds exhibited cell viabilities of 81% for day 1 and 78% for day 3, with scaffold degradation releasing TFA, resulting in lower viability (Figure 4D) and visible scaffold fragmentation along with CS degradation (Figure S6C, Supporting Information). The extracts from the neutralized blend scaffold (nCS+PCL) had a similar effect on cell viability, with values of 84% and 74% for days 1 and 3, respectively, likely due to scaffold degradation effects, even though material fragmentation and degradation were significantly limited (see Figure 4E; Figure S6D, Supporting Information). Further analysis revealed that the extracts from the gCS+PCL scaffold demonstrated a cell viability of 68% for day 1, which improved to 82% for day 3, with the fibrous structure of the scaffold remaining intact (Figure 4F).

Overall, gCS and gCS+PCL showed slower degradation than uCS, uCS+PCL, nCS, and nCS+PCL. It was demonstrated by the presence of degradation and fiber fragmentation products released to the cell culture medium from uCS and uCS+PCL (Figure S6A,C, Supporting Information). In untreated fibers, rapid disintegration occurred in an aqueous medium due to TFA in the structure. Performed neutralization with NaOH in methanol provided some structural stabilization and significantly fewer degradation products were observed in extracts from samples nCS, and nCS+PCL (Figure S6B,D, Supporting Information), while still some morphological changes of fibers swelling could be observed (Figure 4 B and E). The swelling is evidenced by the change in average diameter from 0.33 ± 0.10 µm to 1.7 ± 0.55 µm between uCS+PCL and nCS+PCL, respectively. At the same time, cross‐linked samples maintained unchanged morphology and did not release any degradation products into the cell culture medium (Figure 4C,F). We did not observe any fiber diameter change for the cross‐linked gCS+PCL sample (Figure 1E) with a value of 0.29 ± 0.07 µm and for the sample after 3 days of incubation in cell culture medium (Figure 4F), the measured value was 0.21 ± 0.05 µm. The degradation dynamics observed in this study provide insights into the potential applications of these electrospun fibers. Scaffolds with rapid degradation, such as uCS and uCS+PCL, may be more suitable for short‐term drug delivery applications where a quick release of therapeutic agents is desirable.^[^ ^73^ ^]^ In contrast, scaffolds like gCS and gCS+PCL, which exhibit slower degradation and stable structural integrity of electrospun fibers, are better suited for applications requiring sustained support, such as wound healing, where prolonged integrity can promote tissue regeneration and facilitate the exchange of the dressing for another one.^[^ ^66^ ^]^ These findings highlight the potential to tailor CS‐based scaffold formulations to specific biomedical applications by modifying degradation rates and structural stability through cross‐linking and neutralization methods.

## 3 Conclusion

This study evaluated the potential of CS+PCL scaffolds with variable degradation dynamics for targeted biomedical applications. With SEM imaging, we confirmed consistent fiber morphology of all samples, and mechanical testing showed that PCL enhances the tensile strength of CS‐based scaffolds. Importantly, the cross‐linked with genipin gCS and gCS+PCL scaffolds obtained the most suitable mechanical performance, similar to biological tissues. FTIR analysis confirmed that neutralization and cross‐linking effectively remove TFA solvent residues, stabilizing the scaffold structure. Zeta potential measurements showed that blending gCS with PCL leads to a higher negative charge of −26.27 mV, an essential metric for enhanced cell‐material interactions, drug delivery, and tissue engineering. Our findings highlight that degradation rates, mechanical properties, and biocompatibility can be tuned by providing neutralizing and cross‐linking steps for CS and CS+PCL fibers. Rapidly degrading scaffolds, such as uCS and uCS+PCL, are only suited for short‐term drug delivery, while more stable gCS and gCS+PCL are ideal for wound healing and longer‐term tissue regeneration procedures. As proposed here, CS‐based scaffolds offer tunable properties necessary for biomedical applications requiring specific scaffold stability and functionality.

## 4 Experimental Section

### Preparation of CS, PCL, and CS/PCL Solutions 

Polycaprolactone (PCL, Mw  =  80 kDa) and medium‐molecular‐weight CS (CS, Mw =  190‐310 kDa) were purchased from Sigma‐Aldrich, USA. CS (6 wt%) was dissolved in Trifluoracetic acid (TFA, analytical standard, Roth ≥99,9%, Germany) and Dichloromethane (DCM, analytical standard, Chemland, Poland) solution at 8:2 v:v ratio. Polymer dissolution was carried out with a magnetic stirrer (IKA, Germany) for 4 h at 50 °C with a constant speed of 100 rpm and cooled to RT overnight. DCM was added to the CS/TFA solution before electrospinning and mixed at a speed of 350 rpm. PCL was dissolved in Chloroform (analytical standard, Avantor, Poland) and Methanol (analytical standard, Chemland, Poland) using a 4:1 ratio with a speed of 400 rpm using a magnetic stirrer. To prepare a blend solution, CS (5 wt%) and PCL (15 wt%) were prepared as stated above and mixed to prepare a polymer weight ratio of (1:1) using a magnetic stirrer for 30 min at 250 rpm.

### Electrospinning

All electrospun scaffolds: uCS, PCL, and uCS+PCL blend were prepared using electrospinning equipment with a climate control system (IME Electrospinning, Waalre, The Netherlands). The parameters are listed in **Table** **2**. 

**Table 2:** Polymer solution and electrospinning parameters of uCS, PCL, and uCS+PCL blend electrospun scaffolds.

Sample	Concentration of polymer [w/w] [%]	Applied voltage [kV]	Flow rate [mL/h]	Distance [cm]	Relative humidity [%]	Temperature [°C]
uCS	6	16	0.5	15	40	25
uCS+PCL	5 / 15	15	1.2	13	25	25
PCL	8	16	3.5	16	30	30

### Genipin Cross‐Linking of uCS and uCS+PCL Fibrous Scaffold

Cross‐linking was carried out in two steps: 1) neutralization and 2) genipin cross‐linking. Cross‐linking was performed in two steps: neutralization and genipin cross‐linking. For neutralization, the electrospun scaffolds (thickness: 98.1 ± 8.7 µm for uCS and 64.6 ± 14.8 µm for uCS+PCL) were placed in 18 × 18 mm plastic frames and fully immersed in 20 mL of neutralization solution (1M NaOH in methanol, pure P.A., Stanlab, Poland) for 30 min. The samples were then rinsed with 2 mL of PBS (without Ca and Mg, Adlab, Poland), washed 3 times with DI water, and dried at room temperature (RT). Once dried, the scaffolds were removed from the frames and cut into shapes required for subsequent experiments, resulting in neutralized samples (nCS and nCS+PCL). For cross‐linking, the neutralized samples were not dried before the process but were directly immersed in 20 mL of 0.25% genipin solution (M_w_ = 226.23 unit, Glentham Life Sciences, United Kingdom) for 24 h in a 60 mm Petri dish. After cross‐linking, the samples were washed 3 times with DI water and soaked in fresh DI water for an additional 24 h. The cross‐linked samples (gCS and gCS+PCL) were then dried at room temperature (RT), removed from the frames, and prepared for experimentation.

### Physicochemical Analysis of Scaffold—*Morphology of Fibrous Scaffold*

uCS, gCS, uCS+PCL, gCS+PCL, and PCL fibrous scaffolds were imaged with the SEM (Merlin Gemini II, Zeiss, Germany) using an SE detector, with a current of 150 pA, acceleration voltage of 2.5 kV, and a working distance of 4 mm. Prior imaging the samples were coated with an 8 nm Au layer using a plasma coater (Q150RS, Quorum Technologies, UK). The average diameter of the fibers was measured from the SEM images using ImageJ (NIH, v1.54, USA). The average value was determined from 100 measurements. 

### Physicochemical Analysis of Scaffold—*Wettability Evaluation*

The surface wettability measurements of the contact angle on the nanofibers, uCS, gCS, uCS+PCL, gCS+PCL, and PCL were conducted at room temperature. The volume of 3 µL deionized water (Spring 5UV purification system, Hydrolab, Poland) droplets were dropped onto the surface of each material, and the images are taken using a Canon EOS 700D camera with EF‐S 60 mm f/2.8 Macro USM zoom lens. MB ruler software (version 5.3, USA) was used to measure the contact angles. The average water contact angle was obtained from measurements of five droplets per sample.

### Physicochemical Analysis of Scaffold—*Attenuated Total Reflectance Fourier Transform Infrared Spectroscopy (ATR‐FTIR)*

The ATR‐FTIR measurements of CS flakes, PCL granules, uCS, gCS, uCS+PCL, gCS+PCL, and PCL scaffolds were performed to study the materials' chemical composition. (FTIR, Nicolet Is5, Thermo Fisher, USA). For each sample, the spectrum ranged between 4000 and 400 cm^−1^ was obtained by the conglomeration of 64 scans with a resolution of 4 cm^−1^.

### Physicochemical Analysis of Scaffold—*Differential Scanning Calorimetry (DSC)*

The thermal analysis was performed using DSC (Mettler Toledo, Switzerland), which allowed for the examination of the structure of the CS flakes, PCL granules, uCS, gCS, uCS+PCL, gCS+PCL, and PCL fibrous scaffold. The given results present the average values taken from the three individual measurements of each scaffold. The scaffolds were heated from −45 to 275 °C at 10 K min^−1^. Measurements represent only the first heating cycles and the crystallinity was calculated using Stare software (Mettler Toledo, Switzerland) based on the melting enthalpy of 100% crystalline PCL taken as 139.5 J g^−1^.

### Physicochemical Analysis of Scaffold—*Zeta Potential*

An electrokinetic analyzer (SurPASS 3, Anton Paar, Austria) was utilized to analyze the zeta potential via the streaming voltage measurement conducted for gCS and gCS+PCL samples. The fibers were placed in the cylindrical cell dedicated to porous samples. Before measurement, the samples were soaked in water for 5 min to determine whether shrinkage would occur. Then, the fiber mats were placed in the measuring cell and washed with a 0.01 m KCl solution at a pH of 7, as determined for the measurement by the progressive addition of 0.05 m NaOH. The permeability index obtained during electrolyte flow was ≈140 [−].  The zeta potential was measured four times for both samples at the given pH.

### Physicochemical Analysis of Scaffold—*Mechanical Properties of Scaffolds*

The fibrous scaffolds cut into 2  × 1 cm rectangles were used for tensile testing using a dedicated tensile stage (20N Cell, Kammrath & Weiss, Germany) at T  =  24 °C and RH  = 40%. To protect the sample from slipping, it was secured with double‐sided tape placed in the clamps, and the extension speed of 25 µms^−1^ was applied. The thickness of scaffolds was examined using a light microscope (Axio Imager M1 m, ZEISS, Germany). The thickness values were taken from 5 separate measurements (data shown in Table S1, Supporting Information), and an average value of each sample was used to calculate stress‐strain values. 

### In‐Direct Cytotoxicity Evaluation and Scaffold Stability—*Indirect Cytotoxicity Test*

Indirect cytotoxicity assessment of the scaffolds was done to evaluate any potential release of degradation products that could reduce cell growth. Fibrous scaffolds (uCS, nCS, gCS, uCS+PCL, nCS+PCL, gCS+PCL, and PCL) were cut into circles with a diameter of 15 mm to ensure the same material surface to be exposed to the cell culture medium. The scaffolds was sterilized before the biological studies by immersion in 70% ethanol for 30 min, washing with sterile phosphate buffer saline (PBS, Adlab, Poland), and exposure to UV light for 30 min for both sides of the scaffold. Subsequently, two replicates of each sample type were submerged in a cell culture medium and incubated in the CO_2_ incubator (Memmert, GmbH Co., Schwabach, Germany), at 37 °C, and the extracts from samples incubation were collected after 1 and 3 days. The cell culture medium was prepared with Dulbecco's Modified Eagle Medium (DMEM, Thermo Fisher Scientific, US), supplemented with 10% of fetal bovine serum (FBS, Biological Industries, Israel), 2% antibiotics (penicillin/streptomycin, Biological Industries, Israel), 1% amino acids, and 1% L‐Glutamine (Sigma Aldrich, UK). Additionally, nCS, gCS, nCS+PCL, gCS+PCL samples after 3 days of incubation in a cell culture medium were washed with PBS and air dried. Dry samples were gold coated and imaged by SEM, as previously described in Section 4.4.1.

The NIH 3T3 murine fibroblast cell line (NIH‐3T3, Sigma Aldrich, UK) was seeded in a 96‐well plate at a concentration of 2 × 10^4^ cells per ml and volume of 100 µL for an indirect cytotoxicity study. After 24 h of initial cell adhesion and spreading the cell culture medium was replaced with the extracts from scaffolds incubated for (1 and 3 days), and three repeats from each sample were done. Cells exposed to extracts from samples: uCS, nCS, uCS+PCL, nCS+PCL were imaged by light microscopy (Leica DMi1, Germany) to visualize the presence of degradation products from different scaffolds. The complete medium was used as a control to scaffold extracts, and for inducing cytotoxic conditions, a complete medium supplemented with 20% DMSO was used. After 24 h, an assessment of the potential cytotoxicity of the scaffolds was conducted. The extracts from scaffolds were removed and replaced with a medium containing 20% of CellTiter‐ Blue reagent (Promega. Madison, Wisconsin, United States) Following 4 h incubation, from each well, 100 µL of media with reagent was transferred to a 96‐well plate, and fluorescence was read at 560/590 nm using the microplate reader (GloMax Discover System, Promega, Madison, Wisconsin, United States).

### Statistical Analysis

The statistical analysis was accomplished in OriginPro (ver. 2020b, USA). The errors are based on standard deviation. In cell studies, a one‐way ANOVA, followed by Tukey's post‐hoc, was used for statistical examination, and values were significantly different when *p* < 0.05.

## Conflict of Interest

The authors declare no conflict of interest.

## Author Contributions

The manuscript was written with contributions from all authors. All authors have approved the final version of the manuscript. U.S. performed the conceptualization. U.S., P.S., J.E.K., and N.L. performed the methodology. U.S., P.S., and J.E.K. performed formal analysis. P.S., J.E.K., N.L., M.P., and J.K.K. performed investigation and experiment. U.S., P.S., J.E.K., and N.L. performed data curation. U.S., N.L., and P.S. writing original draft preparation. U.S., P.S., J.E.K., and M.P. performed writing review and editing., U.S. performed supervision, project administration, and funding acquisition. All authors provided critical feedback and helped with the research, data analysis, and manuscript.

## Supporting information



Supporting Information

Supplemental Video 1

## Data Availability

The data that support the findings of this study are available from the corresponding author upon reasonable request.
