# Supplementary material for: Improving Stability and Mechanical Strength of Electrospun Chitosan‐Polycaprolactone Scaffolds Using Genipin Cross‐linking for Biomedical Applications
Source: Macromol Rapid Commun. 2024 Dec 27;46(13):2400869. doi: 10.1002/marc.202400869 (PMC12227225; doi:10.1002/marc.202400869)
Supplement: Supplementary file 1 — Supporting Information [file MARC-46-2400869-s001.docx]

Supporting Information to

**Improving Stability and Mechanical Strength of Electrospun Chitosan-Polycaprolactone Scaffolds Using Genipin Cross-linking for Biomedical Applications**

*Nagalekshmi Uma Thanu Krishnan Neela^1^, Piotr K. Szewczyk^1^, Joanna Karbowniczek^1^, Martyna Polak^1^, Joanna Knapczyk-Korczak ^1^, Urszula Stachewicz^1*^*

*^1^ AGH University of Krakow, Faculty of Metals Engineering and Industrial Computer Science, al. A. Mickiewicza 30, 30-059 Krakow, Poland*

**E-mail: ustachew@agh.edu.pl*

**Video 1** – showing wetting of all the samples uCS, gCS+PCL, PCL, uCS+PCL, gCS with water droplet.

**Table 1.** The average thickness with the standard derivation of the cross-linked and uncross-linked fibrous scaffold.

| **Group** | **Average thickness (µm)** |
| --- | --- |
| uCS | 98.1 ± 8.7 |
| gCS | 172.4 ± 2.0 |
| PCL | 37.3 ± 5.0 |
| uCS+PCL | 86.3 ± 2.4 |
| gCS+PCL | 64.6 ± 14.8 |


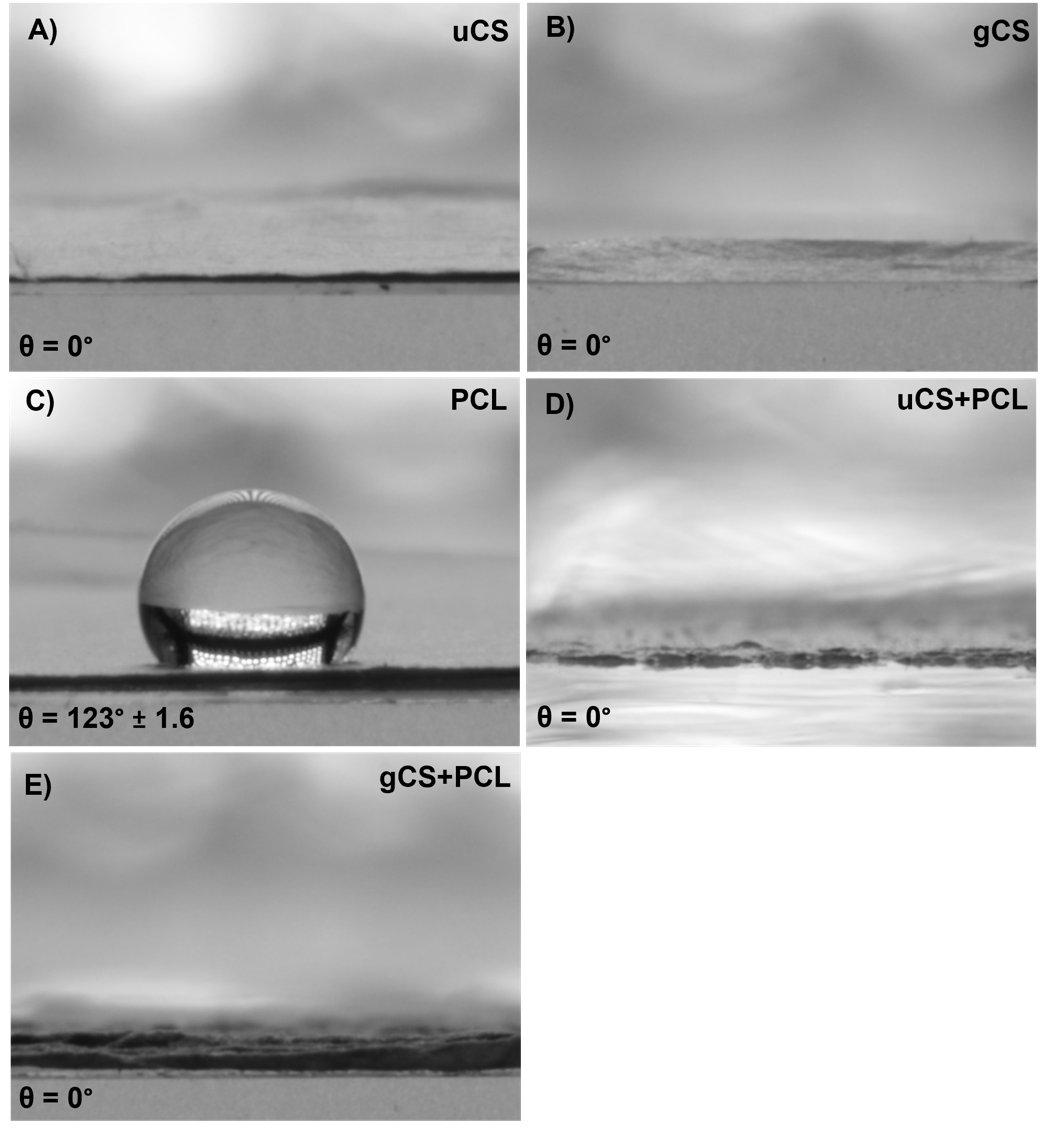


**Figure S1.** Water droplets photograps immediately after deposition on the electrospun scaffold indictaing the wettability of : A) uCS, B) CS, C) PCL, D) uCS+PCL, and E) CS+PCL.


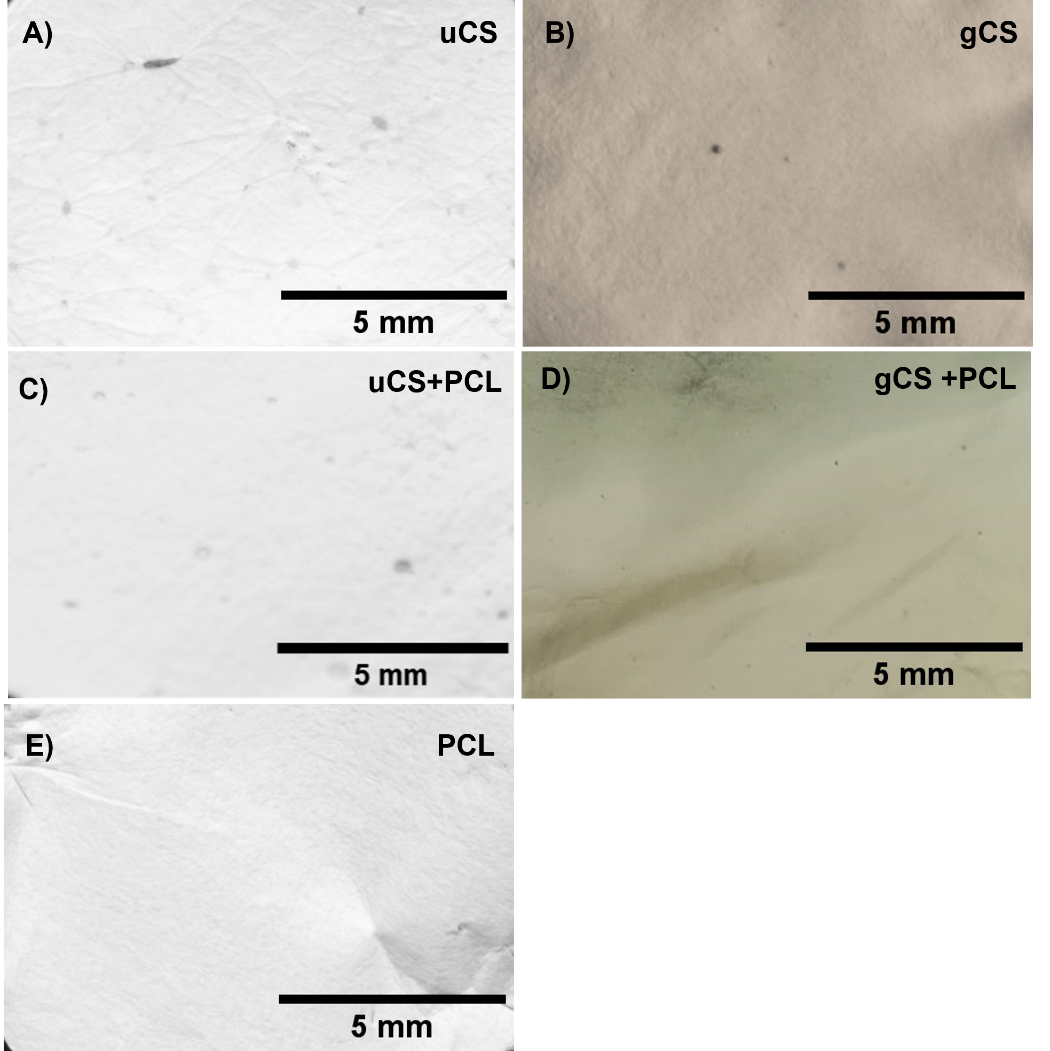


**Figure S2.** Top view photograps showing the gross morphology of the electrospun scaffold: A) uCS, B) gCS, C) uCS+PCL, D) gCS+PCL, and E) PCL.


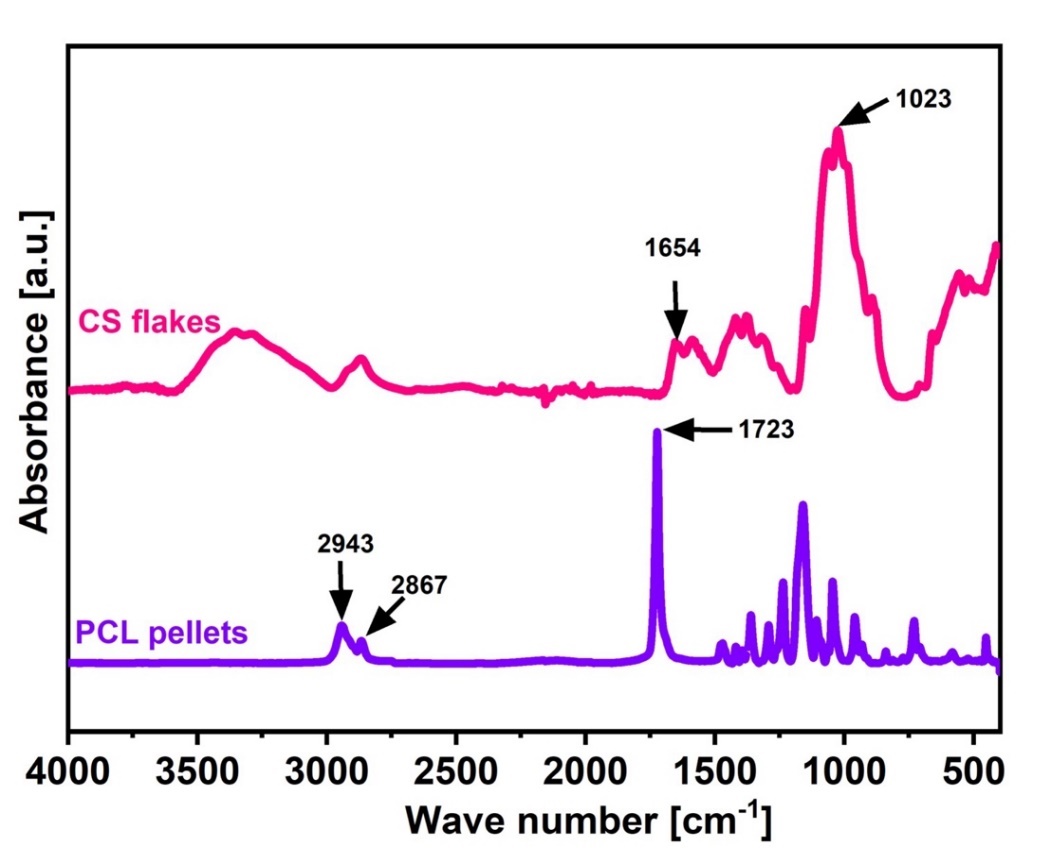


**Figure S3.** The FTIR spectra of electrospun scaffold CS flakes and PCL pellets.

**
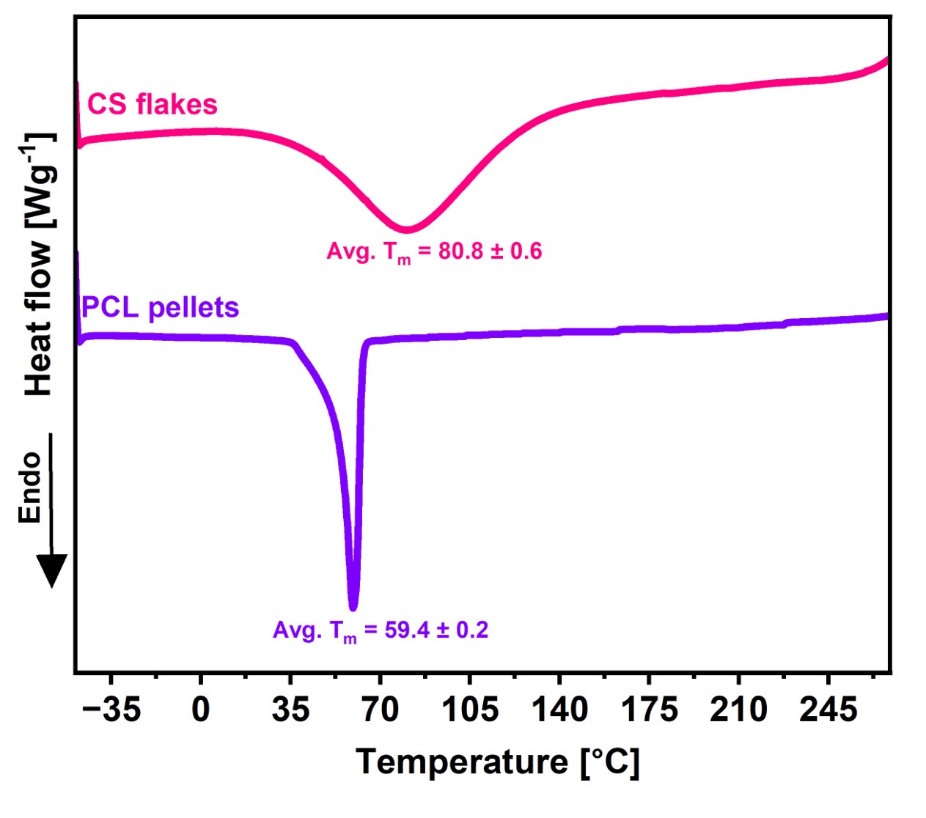
**

**Figure S4.** DSC curves of CS flakes and PCL pellets.


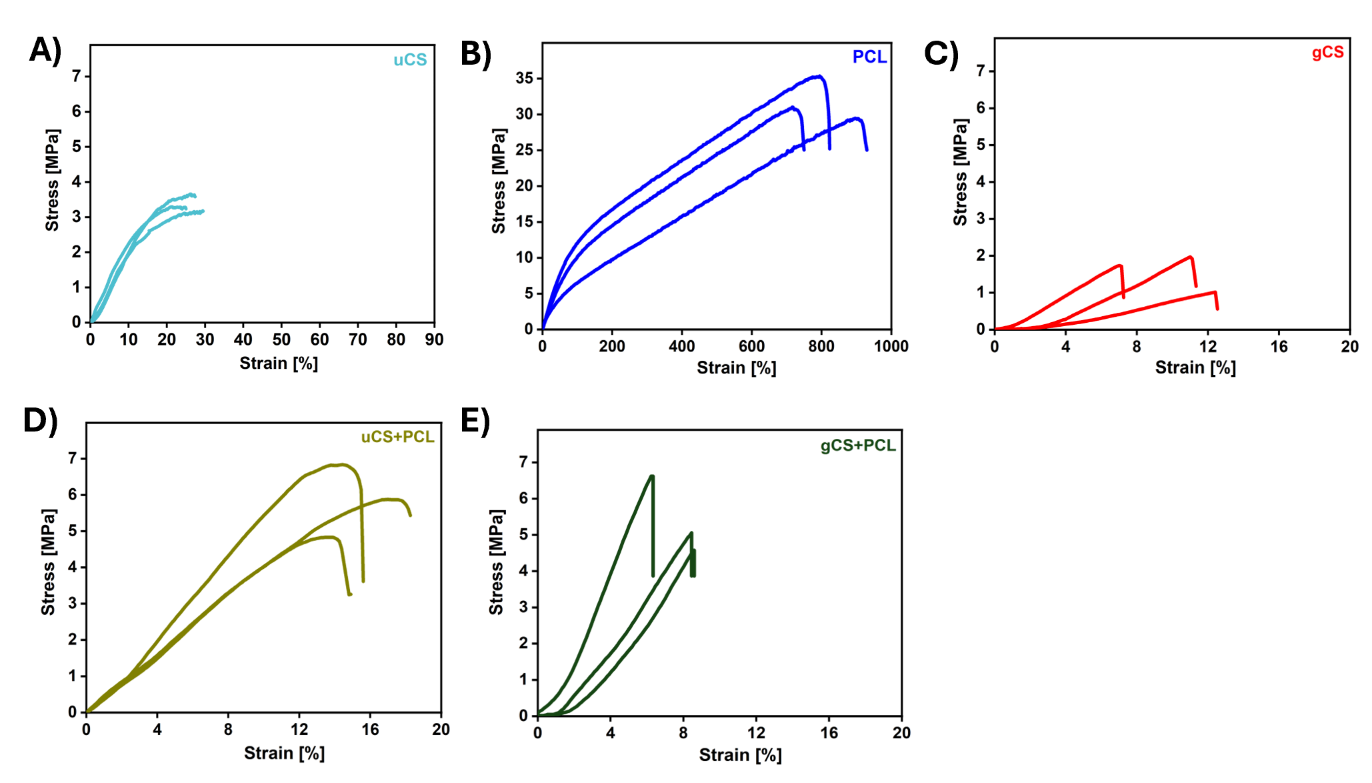


**Figure S5.** Mechanical behavior of electrospun scaffolds. Stress-strain curves from the tensile testing of ranonly oriented electrospun fibers in mats A) uCS, B) PCL, C) gCS, D) uCS+PCL and E) gCS+PCL.


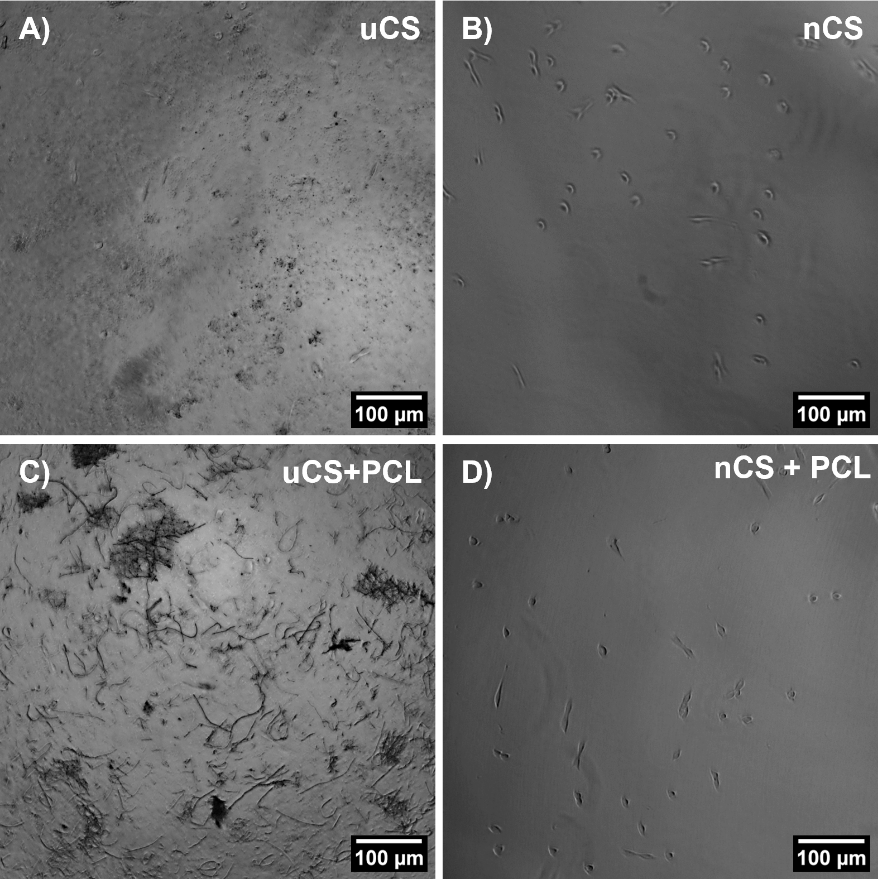


**Figure S6.** Light microscopy images of the NIH 3T3 cells exposed to extracts from scaffolds incubation in cell culture medium A) uCS, B) nCS, C) uCS+PCL and D) nCS+PCL.
